# Between Action and Emotional Survival During the COVID-19 era: Sensorimotor Pathways as Control Systems of Transdiagnostic Anxiety-Related Intolerance to Uncertainty

**DOI:** 10.3389/fpsyt.2021.680403

**Published:** 2021-07-29

**Authors:** Sari Goldstein Ferber, Gal Shoval, Gil Zalsman, Mario Mikulincer, Aron Weller

**Affiliations:** ^1^Psychology Department and Gonda Brain Research Center, Bar-Ilan University, Ramat Gan, Israel; ^2^Geha Mental Health Center, Petah Tiqva, Israel; ^3^Sackler Faculty of Medicine, Tel Aviv University, Tel Aviv, Israel; ^4^Princeton Neuroscience Institute, Princeton University, Princeton, NJ, United States; ^5^Division of Molecular Imaging and Neuropathology, Department of Psychiatry, Columbia University and New York State Psychiatric Institute, New York, NY, United States; ^6^Interdisciplinary Center (IDC) Herzliya, Baruch Ivcher School of Psychology, Herzliya, Israel

**Keywords:** COVID-19, sensorimotor, anxiety disorders, ANS, ascending activating system, descending activation, control theory

## Abstract

**Objectives:** The COVID-19 pandemic and aligned social and physical distancing regulations increase the sense of uncertainty, intensifying the risk for psychopathology globally. Anxiety disorders are associated with intolerance to uncertainty. In this review we describe brain circuits and sensorimotor pathways involved in human reactions to uncertainty. We present the healthy mode of coping with uncertainty and discuss deviations from this mode.

**Methods:** Literature search of PubMed and Google Scholar.

**Results:** As manifestation of anxiety disorders includes peripheral reactions and negative cognitions, we suggest an integrative model of threat cognitions modulated by sensorimotor regions: “The Sensorimotor-Cognitive-Integration-Circuit.” The model emphasizes autonomic nervous system coupling with the cortex, addressing peripheral anxious reactions to uncertainty, pathways connecting cortical regions and cost-reward evaluation circuits to sensorimotor regions, filtered by the amygdala and basal ganglia. Of special interest are the ascending and descending tracts for sensory-motor crosstalk in healthy and pathological conditions. We include arguments regarding uncertainty in anxiety reactions to the pandemic and derive from our model treatment suggestions which are supported by scientific evidence. Our model is based on systematic control theories and emphasizes the role of goal conflict regulation in health and pathology. We also address anxiety reactions as a spectrum ranging from healthy to pathological coping with uncertainty, and present this spectrum as a transdiagnostic entity in accordance with recent claims and models.

**Conclusions:** The human need for controllability and predictability suggests that anxiety disorders reactive to the pandemic's uncertainties reflect pathological disorganization of top-down bottom-up signaling and neural noise resulting from non-pathological human needs for coherence in life.

## Introduction

The human mind seeks coherence in life (1). The COVID-19 outbreak exposed the world to a prolonged uncertain situation which in turn poses a risk for increased psychopathology in the general population across ages and increased risk in individuals with pre-existing psychiatric disorders (2–6). A neglected aspect of this pandemic is the mental health suffering of the whole population (people who lost their relatives, out of jobs, familial economy crashes, people confined at home, elder people socially isolated and the worst, uncertainty about the future). Calls for proactive approaches to treat have been published (7).

Anxiety disorders are associated with intolerance of uncertainty (8, 9). The highly anxiety-related concept, intolerance of uncertainty, has been historically defined in science in the early 1990s and further developed into a transdiagnostic risk factor suggesting that intolerance to uncertainty fuels reactive anxiety across many disorders (10) and this is evidence-based (8, 11). Anxiety disorders have the highest prevalence of all mental disorders, displaying compromised functioning with varied levels of prognosis and remission (12). In addition, treatment resistance or partial remission are prevalent (13, 14).

The objective of this review is to identify the brain circuits involved in the human reaction to uncertainties and their connectivity with sensorimotor pathways. Additionally, this review aims to suggest treatment implications for increase of the tolerance to uncertainty based on the identified circuits and their motor counterparts. Our view is of major importance for timely treatment while facing the secondary effects of the COVID-19 outbreak, which is the peaking pandemic of mental health.

The neuroscience of the healthy individual or “the confident brain” shows that in situations of uncertainty distinct neural populations actively compute, in a stepwise manner, the chances for a rewarding experience and the potential costs and create an anticipation which is the guide for adaptive behaviors. This develops from (1) sensory processing, (2) internal state and environmental evaluation, (3) deciding on constancy of input variables, comprising a rule of certainty or uncertainty, and finally (4) anticipating an outcome prediction that results in particular action to be taken. This process involves the human capacity for self–control, which is conceived as the last, active part of the capacity for self-regulation. Self-regulation according to this approach has been defined (15, 16) as the ability of the organism to return to baseline after mounting specific responses to an environmental stimulus. Intrapersonal neurobehavioral co-regulation is defined as the capacity of the organism to subordinate all neurobehavioral capacities to enhance learning that allows it to be adaptive to the environmental requirements. It is also defined as the capacity of the organism to return to balance, following adaptation of the enhanced neurobehavioral subsystem to the environmental stimuli (16, 17). The operational definition of self-control includes everything that one does in the “operate” phase, which is the “return to balance” phase in the definitions of self-regulation. This is in accordance with classical and well-accepted theories on the human subjective sense of perceived control (18) and the need for predictability to establish and maintaining control (19). The process for coping with uncertainty described above also involves aspects of anticipated cost and reward (20) within the capacity for goal conflict regulation (21).

Goal conflict regulation has been found to be decreased in conditions of anxiety and depression (22, 23), suggesting that this capacity may enhance self-control by optimizing predictability and the pathways of cost reward calculations. Thus, self-control, the sense of controllability and calculations of cost and reward through the global function of goal conflict regulation are involved in the healthy process of coping with uncertainty. This has been related to facilitation of the Behavioral Inhibition System's (BIS) (24) reaction by supporting predictability toward making a choice and taking an action. Interestingly, hippocampal and right frontal theta frequencies have been suggested as biomarkers of “healthy anxiety” arising from situations of goal conflict and uncertainty suggesting different types of arousal are responsible for the generation of anxiety in healthy individuals (21, 25, 26). Higher frequencies such as alpha rhythmicity have been recently reported as appearing at the last stage of the process of goal conflict regulation and suspected as a result of the motor system (27). Anxiolytic action on the behavioral inhibition system implies that multiple types of arousal contribute to anxiety. Accordingly, we argue in this paper for the centrality of motor regions in regulating healthy cognitive processes of uncertainty. It has also been found that the anxiety related to the theta frequency has not been captured by anxiety and depression as assessed by well-accepted inventories (27).

We suggest in this paper a process related to healthy coping and supposedly “healthy anxiety” (21, 24, 28), which in turn may serve as a lesson for treatment of more pathological cases of anxiety. The centrality of motor regions in encoding and resolving situations of uncertainty is implicated in the existence of a goal or multiple goals requiring an action to be taken as suggested in the global function of goal conflict regulation. The difference between healthy and pathological anxiety in uncertain situations is suggested to be embedded in the existence of goals to be regulated and more than that, in the availability of realistic goals to be achieved and that an action toward one of them matters for the resolution of uncertainty. The Sensorimotor-Cognitive-Integration-Circuit (SCIC), described below, is suggested to be the basis for healthy processing of uncertainty.

In the last section of this paper, we suggest the centrality of sensorimotor regions as a target for psychotherapeutic interventions aimed to increase confident cognitions in anxiety disorders during the COVID-19 era.

## Intolerance to Uncertainty in Anxiety Disorders

Regular daily life consists of different aspects of certainty and uncertainty. The human mind utilizes anticipation in order to accommodate to new and uncertain situations. In anxiety disorders, this positive anticipation allowing adaptive behaviors is at least partly impaired. A state of intolerance to uncertainty is a core factor in anxiety psychopathology (29, 30). We suggest that prolonged situations of uncertainty such as the COVID-19 pandemic may increase the risk of and prevalence for developing anxiety disorders in the general population.

Higher levels of intolerance of uncertainty are associated with internalizing psychopathology, including generalized anxiety disorder, obsessive compulsive disorder, social anxiety disorder, panic disorder, depression, and eating disorders (29, 31). Across disorders, uncertainty is thought to provoke anticipatory anxiety and to result in behaviors that are maladaptive attempts to reduce uncertainty, such as worry, reassurance seeking, checking, and hypervigilance (32, 33). In many cases of anxiety disorders, a constant perception of uncertainty without environmental justification is prominent. In others, on which this review focuses, environmental uncertainty elicits, or exacerbates the onset and reoccurrence of anxiety symptoms.

Anxiety disorders are characterized by a wide range of cognitive and somatic symptoms and sufferers have a higher lifetime prevalence of overall dysfunction and co-morbid psychopathology, particularly depression. Lifetime rates of cardiovascular, respiratory, gastrointestinal, and other medical problems are disproportionately high in individuals with anxiety and panic/fear disorders (34). Epidemiological survey findings show that anxiety disorders are the most prevalent mental disorders worldwide and they are associated with significant comorbidity and morbidity (35, 36). The WHO recently estimated global mean prevalence for any anxiety disorder, including PTSD, as 21.7% in the general population (12). Treatment resistance is reported to be also very prevalent (13, 14).

We present our view of anxiety-related behavioral, somatic and subjective manifestations from a transdiagnostic perspective. This is supported by the view adapted in the DSM-5 (37) in which the five supposedly distinct axes of the DSM-IV were deleted and the new approach published in 2013 requires the indication of the levels of disorders' severity and need for outside facilitation. In the DSM-IV, anxiety disorders were related mainly to axis 1 although detected in disorders included in axis 2 too. The approach taken by the DSM-5 advises that elicitation and reoccurrence of anxious reactions may appear in a transdiagnostic manner.

We propose that anxiety reactions are a continuum ranging from “healthy anxiety” to pathological anxiety which may be termed as “the anxiety spectrum.” The healthy anxiety is defined by reacting to an objective threat with defensive alarmed behavioral and subjective responses which are justified by the existence of the threat (21, 24, 28). Pathological anxiety depends on the severity of the reaction and the level of negative impact on regular functioning according to the DSM-5.

The transdiagnostic approach has been recently suggested as validated by treatments which were generally outlined and currently prescribed for a wide range of patients with considerable therapeutic broad effects (28, 38, 39). Additionally, the results of the National Comorbidity Survey (40) which was an epidemiological study, with 65,244 adult participants in the United States aged 15–54 years suggest a small number of “pure” cases and revealed a majority of comorbid cases. Furthermore, this approach determines fewer stigmatic effects and implies a humanistic perspective of a particular individual condition.

We also suggest that pathological anxiety presents a reaction to internal cues, which cause over-arousal and hypervigilance to external cues, not necessarily encoded as a detailed cognition. This view puts forward the pathological matching of internal and external cues in severe cases, suggesting that uncertain situations are transformed into a threat perception by negative anticipations. Furthermore, our transdiagnostic approach is in accordance with the “transdiagnostic model of uncertainty” (28, 39, 41, 42), which suggests that a threat perception is generated when one is confronted with an uncertain situation. In accordance with recent evidence (8, 10, 11) we also suggest that the matrix of the intolerance to uncertainty axis by the anxiety reactions axis builds up to extremity and severity via bi-directional effects of intolerance to uncertainty on anxious reactivity and vice versa.

From a cognitive perspective, people with anxiety disorder translate the ambiguous situation into a threat, thus the top-down bottom-up cerebral connectivity produces a fear reaction (43). From a neurochemical angle a stress reaction is recruited resulting from the fear cognition and the threat perception (44–46). From a peripheral perspective, sympathetic over-arousal is apparent including peripheral reactions such as psychogenic tremor, perspiration, increased heart rate, respiration and gastrointestinal symptoms (47).

Neural noise {the random intrinsic electrical fluctuations within neuronal networks which are not associated with encoding a response to internal or external stimuli; [e.g., (48, 49)]} has been suggested to be involved in the reactions of individuals with anxiety disorder to uncertainty (30, 50, 51). Neural noise may be the basis of an exaggerated anxiety reaction. Neural noise may result from over-reaction to alarming peripheral cues without a clear cognitive estimation or evaluation of a threat. Therefore, competing negative signals to the cortex may not be processed accurately in a condition in which an internal peripheral alarming cue meets external potentially threatening stimuli and this assembly combat in neural circuits occurs without a time limited resolution within the cortical descending order. This may be part of the sympathetic reactivity and the basis for the peripheral symptoms (52).

In this respect, neural noise represents the competition between multiple intrinsic and extrinsic driven signals, which carry various types of negative information. This may block the cortical proficiency to attribute a clear cause to the signals thus no goal-oriented resulting decision on an action to be taken is available as the facilitating encoding is lacking. It is predominantly a neurological state encompassing a reduction in cortical production of encoded cognitions. In uncertain situations, the salience of a general negative experience generated by internal somatosensory systems produces neural noise which may also block the availability of other sensory inputs as they become available, including signals which carry relaxing information, thus increasing and prolonging, even fixating, the risks for anxious reactions. Thus, the subjective sense of anxiety in uncertain situations may be experienced as objectively driven even though internal cues are heavily involved and objective threats may be overly evaluated as costly in this condition.

We suggest a model of complex brain cyclicity, emphasizing the role of sensorimotor regions in the generation of certainty vs. uncertainty cognition. Of special interest of this review is the issue of autonomic nervous system (ANS) coupling with the cortex to address peripheral reactions of anxiety disorders to uncertainty beyond a simple distorted cognition as widely accepted.

## A Biological Model for the “Confident Brain”

**Figure 1:**
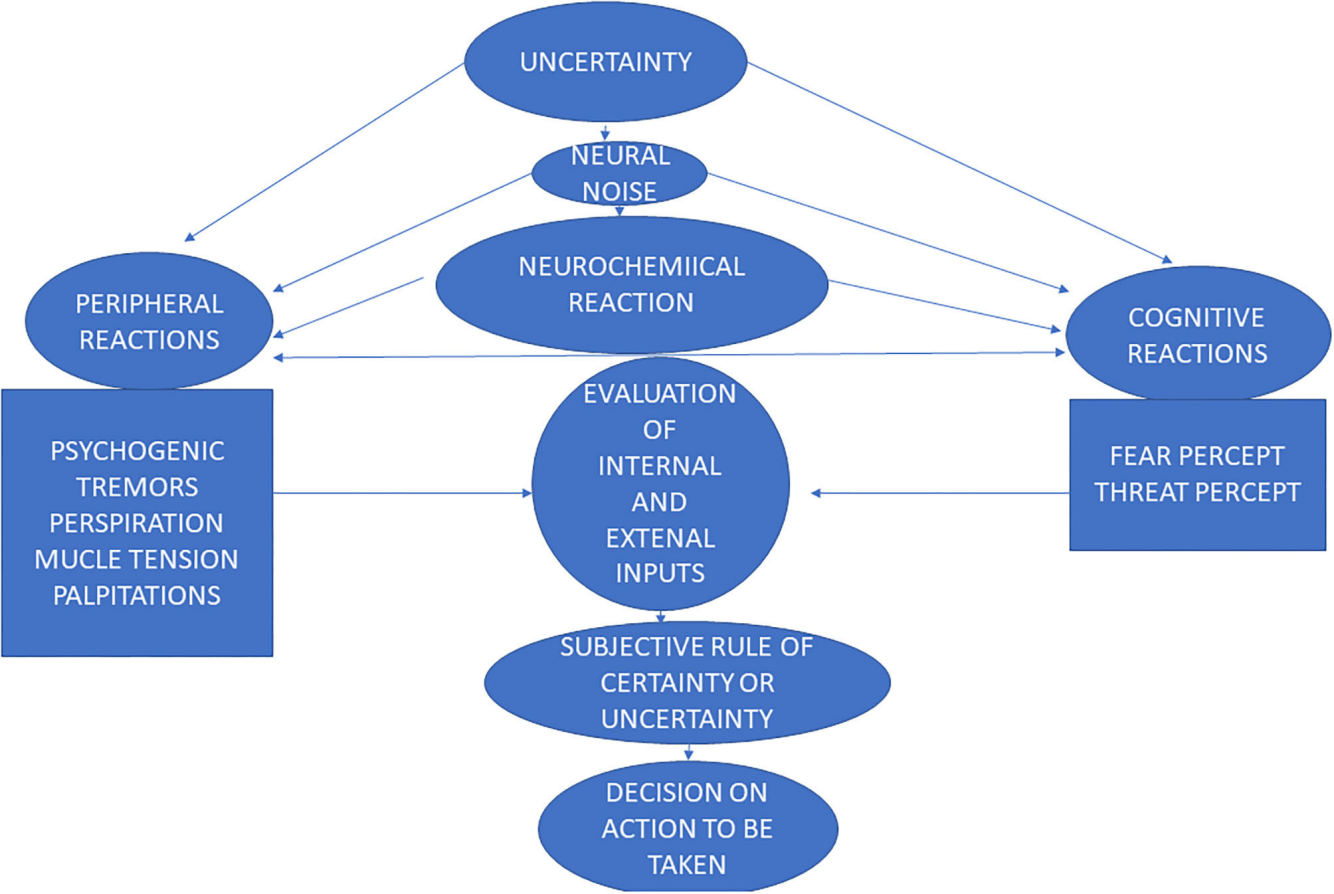
The process of uncertainty from the stage of sensory input to the stage of making a decision on an action to be taken, in anxiety disorders.

The healthy cognitive coping with uncertainty or the “confident brain” employs a stepwise process using (1) sensory processing; (2) state and environmental evaluation; (3) recognizing whether a rule of certainty exists or rather a rule of uncertainty and finally (4) outcome prediction (50), see Figure 1. Recent data show that this process of uncertainty evaluation and resulting reaction involves top-down and bottom-up cyclical brain modulation in numerous regions, including sensorimotor regions, the limbic system, the reward circuits and frontal regions responsible for executive functions (53, 54).

From a neurochemical perspective, noradrenaline influences learning of uncertain events arising from unexpected changes in the environment (55). In contrast, acetylcholine balances attribution of uncertainty to chance fluctuations within an environmental context, defined by a stable set of probabilistic associations, or to gross environmental violations following a contextual switch (55). Dopamine supports the use of uncertainty representations to engender fast, adaptive responses (55). Diurnal cortisol is disrupted in prolonged uncertain situations (56) and in anxiety disorders (57). Various studies support the role of dopamine and serotonin in anxiety disorders (58–60).

From a peripheral perspective, healthy participants reacted to uncertainty with altered spinal reflexes (30, 61) and gastrointestinal symptoms (62–64). Uncertainty related sympathetic over-arousal has also been indicated (52, 65).

**Figure 2:**
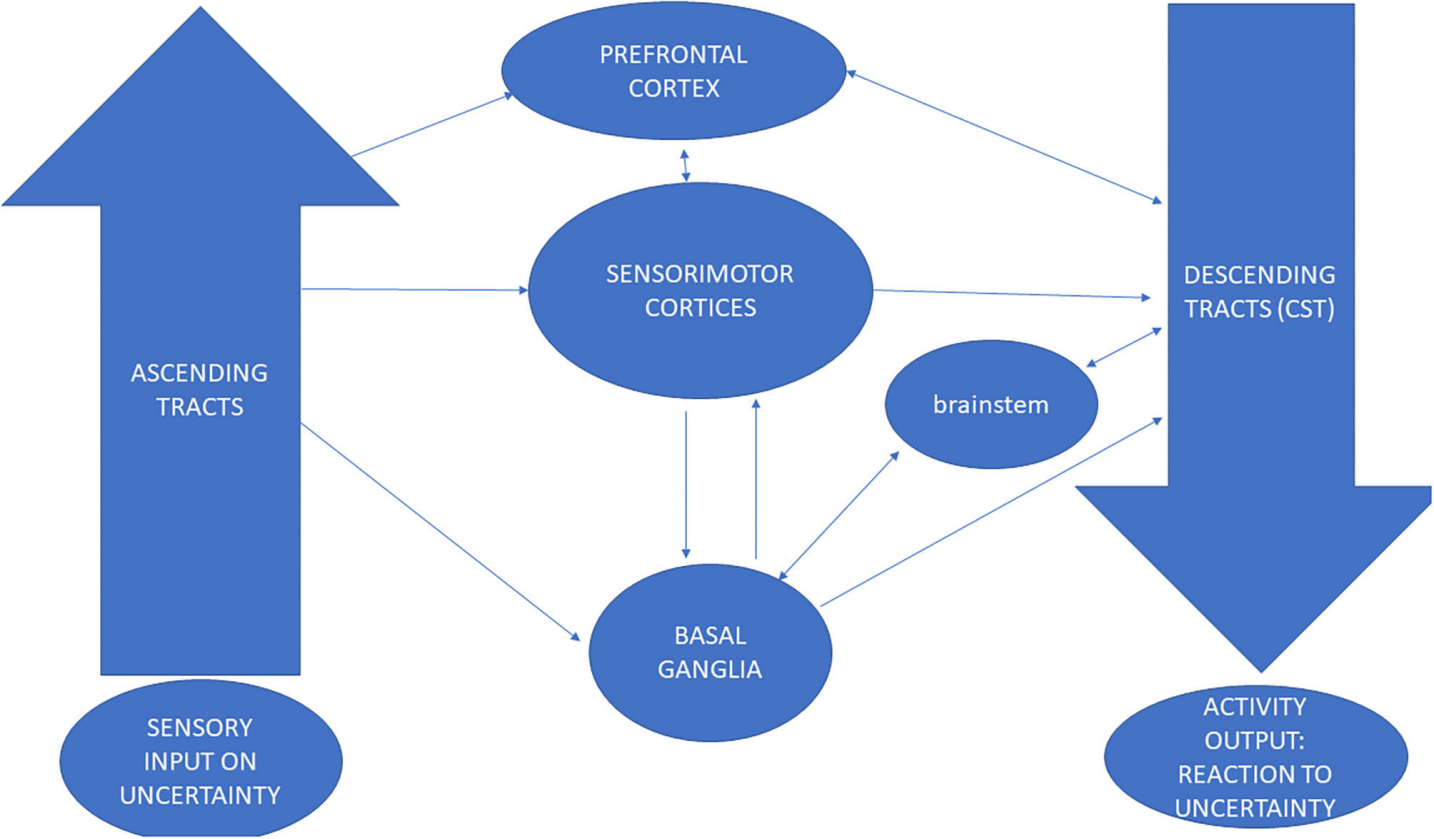
The involvement of sensorimotor circuits in processing and filtering uncertainty cognitions in the healthy brain: The Sensorimotor-Cognitive-Integration-Circuit (SCIC).

The thalamo-cortico-striatal circuit (TCS) is thought to be central in the pathophysiology of anxiety disorders (66) in addition to the amygdala and the corticolimbic system (67, 68). We suggest a more complex circuit for the impact and processing of uncertainty, which is hampered in anxiety-related disorders. Dysregulated coupling of the ANS with the cortex in anxiety disorders (69) may be shown in the compromised function of the tracts of the crus cerebri traveling from the cortex to the spinal cord, filtering information *en passage* (70). The coupling of the autonomous system is also supported by ascending tracts reaching sensorimotor cortices through filtering information by the basal ganglia and amygdalae (71–73). Specifically, microstructural differences in the bilateral corticospinal tracts (CSTs) were detected among patients with anxiety and depression (66, 74). Lower fractional anisotropy (FA) was found in the white matter of the CST. Further, increases in FA in the CST-related fiber paths of the bilateral posterior limbs of the internal capsule, right superior corona radiata, and the left external capsule were found while an increase in CST excitability has been detected in participants exposed to fearful images (75). The CST has been shown to have a central role in a more complex circuit for evaluation of uncertainty and tolerance to ambiguity in anxiety disorders. Changes in gray matter in the basal ganglia and in white matter in the corticospinal tract were reported in anxiety disorders (66). In over-arousal and over-stimulated conditions the corticospinal neurons' function is inhibitory through their affinity to GABAergic and glycinergic presynaptic function (76). Both basal ganglia and the corticospinal tract which were historically accepted as motor regions, have been recently recognized as involved in cortical and subcortical filtering of information that is transmitted to and commanded by the prefrontal and somatosensory regions (77). Thus, the link between sensory integration and motor output, the input and output of healthy processing of uncertainty (50), operate in a top-down bottom-up manner. This involves commands to peripheral regions such as those ending in spinal reflexes as well as posing significant impact on cognitive and emotional processes (72). We suggest that ascending sensory and descending corticospinal tracts are integrated in the somatosensory cortex and that they selectively project to the prefrontal cortex following filtering by the basal ganglia and the amygdalae. It is further suggested that in anxiety disorders these neural connections are hampered (78–82), see Figure 2.

Our model complies with the rules of Control Theory, developed from Maxwell's mathematics (83) and utilized in engineering (84, 85). The first applications to Psychology are found as early as the 1940's in the works of Craik (86), Wiener (87), and Ashby (88). Since the 1990's, Powers' (89) application of Control Theory to human goal-oriented behavior and his development of perceptual control conceptualization termed as Perceptual Control Theory (PCT) has gained renewed scientific attention (90) while previously regenerated in the form of a self-regulation theory, [e.g., (18, 91, 92)] relating to Cannon's view on homeostasis (93) and Wiener's (87) theory on Cybernetics as essential parts of it.

Control Theory implies discrepancy-reducing feedback loops for correction of error signaling by various controllers' sensors in each of the sub-systems which together activate the entire control system. PCT implies a hierarchy of eleven modules, ranging from sensory input “intensity” (28) to personal transcription of “programs,” “principles,” and “systems,” which are the highest modules (94) ending in the organization of the individual's principles in systematic personal organization, while all modules are functioning in a top-down bottom-up manner to elucidate a perception for a goal directed behavior elicitation. These modules maintain feedback loops and error correction signals (termed as internal reference standards) within each loop and each module as well as between modules for an overall perception on a goal directed behavior. Recently this theory has been applied to healthy coping with uncertainty suggesting the “The Effort Intensity Continuum” developing from avoidance to toleration, coping, and embracing. McNaughton and Gray (21), Gray (24), Gray et al. (25), Einstein and Mansell (28), and Gray (95) describe how a central comparator system may trigger a pause in an operating program within multiple trials to resolve the mismatch between the perceived threat (cost) and the individual's desired outcome during defensive approach (expected reward). The term reorganization has been suggested by Powers (89), to be apparent as a trial and-error learning process leading to the resolution of this mismatch conflict in uncertain situations. Carey (96) suggests that this phase of reorganization requires sustained attention by the systems responsible for encoding the goal conflict toward reaching a resolution, which he agrees is a decision on an action to be taken as we argue in this paper.

Our model also is in accordance with the Perceptual Control Theory (PCT) (41) which proposes, in line with the mathematical Control Theory formulations, that the nervous system as a whole is an organized control system in which higher systems receive feedback from lower systems, describing a cascade of levels. Accordingly, we suggest that the neurological pathways identified as central for coping with uncertainty along the central nervous system (CNS), are fed back in a top-down bottom-up manner, within a cyclical control system.

A good example for that is our claim that although sensory inputs start the process of coping, our model suggests that the sensory inputs by the ascending tracts may be transformed or even corrupted (97) in cases of prior cognition, especially if there is a sensory-input-opposing-cognition. Thus, the starting signal may be sensory (bottom-up) in healthy cases and cognitive-distorted (top-down) in more pathological cases of coping with uncertainty including the exaggeration of negative (cost) view of it. Therefore, while processed on the continuum of the feedback loops, a particular sensory input may affect the descending tracks with a deteriorating error implying a destructive, not correctly computed signal. In such a case, the decision on the action to be taken may not be useful for reducing uncertainty. This could potentially explain pathological coping with uncertainty and its aligned increased anxiety.

It is suggested in our model that signals occur “on the fly” throughout the process and contribute to its continuation with a healthy, realistic, and achievable goal of an action to be taken. This is in accordance with PCT's main feature, explicitly modeling the control of sensory input. This is contrary to previous computational models of the mind which imply precise predictions as well as a mindful mapping to support control of the individual's behavior and the outside world prior to an action (41). That is, healthy coping with uncertainty respects sensory inputs as they become available and does not intend to override them with a-priori cognitions. This is modulated by a healthy ANS. However, the role of higher cortical regions such as the sensorimotor areas, in healthy cognition creation for a decision on an action to be taken, is argued here in accordance to the PCT, to be within the circuitry continuum of the multivariate error correcting and feedback processing.

## The Sensorimotor Areas' Connectivity With the Amygdala

Likelihood uncertainty is represented in the associated sensory pathway (visual). In contrast, prior uncertainty is represented in putamen, amygdala, insula, and OFC (98).

Resting state (rs)-fMRI studies show alterations in the amygdala-sensory/(pre)motor pathways and suggest that these alterations may be involved in psychiatric conditions (99). Specifically, others suggest recently that the amygdala and bed nucleus of stria terminalis (BNST) connectivity are neural markers of anxiety disorders. Whereas, amygdala-thalamus/ACC rs-functional connectivity supports adaptive regulation of threat response in healthy controls, BNST-caudate rs-functional connectivity may reflect maladaptive neural processes that are dominated by anticipatory anxiety (100).

## The Sensorimotor Areas and The Filtering of Threat Cognitions By the Basal Ganglia and Amygdala

Classically, the basal ganglia have been considered to have a role in producing habitual and goal-directed behaviors. Recently evidence indicates that the basal ganglia are also involved in neural and behavioral inhibition in both goal-directed and habitual choice-preference motor actions mediated by fronto–striato–subthalamic–pallido–thalamo–cortical networks. Imbalance between goal-directed and habitual action and inhibition has been suggested as involved in manifestations of neuropsychiatric disorders (101). Thus, the inhibitory role of the basal ganglia over motor outputs resulting from reactions to uncertainty, suggests that it filters signals to motor cortices involved in reactions to uncertainty such as the sensorimotor regions. These inhibitory signals may be available to the cortices through excitatory terminals in the striatum (101) raising the hypothesis that competing signals of inhibition (threat cognition) and excitation (reward cognition) are imbalanced in conditions of intolerance to uncertainty, such as evident in anxiety disorders which in turn may end in erroneous expectations and less than evidence-based motor outputs of a decision.

## The Sensorimotor Areas and Cognitions of Reward and Assurance

Recent studies suggest an important role of the basal ganglia in reward expectation (102), a missing component in anxious intolerance of uncertainty. Sensorimotor/cognitive activities of neurons in the basal ganglia are strongly modulated by expected reward. While signaling to the brainstem, the basal ganglia may affect the CST too, which in turn connects the brainstem to the cortices and couples the ANS to them. Thus, peripheral reactions in anxiety conditions resulting from intolerance to uncertainty may be based on dysfunction of the differential signaling of the basal ganglia. Neurons in the caudate nucleus and the substantia nigra pars reticulata are extremely sensitive to differences in expected reward. Therefore, they lead to a bias in excitation/inhibition between the superior colliculi suggesting that motor integration toward a reward could occur more quickly or slower (103). Therefore, these results may explain the approach-avoidance (24) conflict in anxiety conditions when faced with a probability of reward ending in a threat perception and avoidance behavior because of negative interpretations of rewards that are not certain. It is suggested that the reward modulation occurs in the caudate where cortical inputs carrying spatial signals and dopaminergic inputs carrying reward-related signals are integrated. This suggests the centrality of the basal ganglia sensorimotor cognitive neurons in filtering of the competing signals to the motor cortices and through its connectivity all the way to the CST via its signals to the brainstem.

## The “Emotional” and “Affectively Conscious” Brainstem And Its Role in Somatosensory Integration and Motor Output

Affective Neuroscience's approach suggests that affective consciousness is generated and processed by sub-cortical areas (104). Accordingly, the “emotional brainstem” includes three major networks—Ascending, Descending and Modulatory (105–107). The brainstem is a somatosensory-motor integrating gateway for emotional and consciousness regulation. The ascending network includes the spinothalamic tracts and their projections to brainstem nuclei. The Descending motor network includes medial projections from the reticular formation and modulation of signaling impacting emotional salience, and other lateral projections to higher regions such as periaqueductal gray, hypothalamus, and amygdala that integrating behavioral actions resulting from emotional regulation. The brainstem regulatory functions are prominent through neurotransmitter pathways including the serotonergic raphe nuclei expression of serotonergic synapses, the connectivity to ventral tegmental area dopaminergic expression and locus coeruleus synaptic effects of noradrenergic activity. All these neurotransmitters are coordinated through different loci in the brainstem. Phylogenetically older brainstem networks work in a caudal to rostral manner with bidirectional signaling to evaluate sensory information and modulate somatosensory inputs including those which are peripherally generated. Thereafter, the brainstem triggers neurobehavioral action patterns (105–107). Ontogenetically, the brainstem and spinal cord develop within two separated plates parted by the sulcus limitans in the brain's fourth ventricle. This ventricle separates the alar plate, from which sensory neurons emerge and the basal plate, from which the motor neurons arise (108). The CST as the largest connection of the cortex to the brainstem is suggested as the main pathway for integration of sensory input toward an action to be taken.

## Prognostic Consideration of Psychiatric Conditions Resulting From the Covid-19 Situation

Sensorimotor activity in anxiety disorders may be habitual or peripherally dysregulated while goal-directed actions may include mainly activities aimed at reducing the sense of anxiety and increasing the sense of certainty. As previously suggested (21, 41), we argue that goal regulation by error correction aligned to accumulating sensory input characterizes the healthy coping with uncertainty, while goal conflict is typically evident in pathological cases of severe anxious reaction in uncertain situations, in accordance with earlier findings (25, 26). Additionally, we propose that the maladaptive coping with- and intolerance to uncertainty is a condition in which the lack of specific achievable goals is apparent. In this condition, small achievable goals do not reach a high expected rewarding value and the individual is aiming at a global, beyond his/her control, goal. For example, a goal of absolute protection from the pandemic or the wish that the pandemic would be shorter or readily over.

We argue that the lack of specific achievable goals, may result from the predominantly neurological condition of neural noise which prevents a clear cognitive attribution to a cause, thus inhibiting cortical descending order toward a motor output, namely, an action to be taken. This condition precludes a resolution, extending, and exaggerating the anxious reaction to uncertainty. That is, goals defined unclearly imply the diffusion of actions. This diffusion of action in turn, results from the build-up of the intolerance to uncertainty and anxiety matrix (8, 10, 11) into severity and extremity in a bi-directional manner. These anxiety-related diffused actions may become a challenge during the COVID-19 era, which consists of real threats and uncertainties. We aim to suggest a treatment approach which may target the bi-directional pathways of perceptual tendencies in anxiety disorders and sensorimotor modulation to increase the tolerance to uncertainty by the selection of achievable goals prior to working toward a resolution. Selection of goals is considered by PCT an input and as such it is of therapeutic merit (109, 110).

The sense of control is closely associated with reduction in anxious reaction to a given situation (111, 112). The need for general control is based on the human need for coherence in life (1). Thus, we argue that the pathological intolerance to uncertainty exhibited in anxiety-related disorders originates from the non-pathological need for controllability and predictability. This need is highly challenged during the pandemic. We aim to suggest a pathway for stimulating motor actions modulated by positive cognitive inputs, which of course could not control the COVID-19 entire situation but may enhance a partial sense of control and sense of coherence in the anxious individuals' personal life during the pandemic. We will outline manners for reactivation of sensorimotor systems within the limits of predictable outcomes to release the individual from the pathological automatic and habitual motor actions, which lack the resulting sense of control. This will allow filtering of sensory inputs and motor outputs, which may result in a bi-directional mode of cognitive driven motor actions affording partial control and more accurate and positive prediction. As emotional survival is threatened by the entire COVID-19 situation, we suggest that the threatened individual has “always something to do about something” meaning partial control in predictable situations and familiar human circles. This line of defense is supported by results of heightened perceived loneliness during the pandemic which attests to a subjective common experience not necessarily depending on physical loneliness (113).

For prognostic considerations, uncertainty requires analysis and treatment of insecurities in the patient's social support circle and the therapeutic definition of the particular unrewarding experiences and percepts and individual translation of uncertainty. Both acute and chronic anxiety conditions should be targeted from a peripheral perspective too and not only from a top-down brain regional view as human cognition of uncertainty in anxiety disorders is associated with bodily sensations. Regulation of physical sensations may support the rise of positive anticipations in uncertain situations and conditions. In anxiety disorders this could be supported and achieved by short-term techniques targeting control over bodily sensations along with changes in anticipatory cognitions. The combination between social support and sensorimotor control is required as simple correction of a negative expectation in uncertain situations may relapse with potential reoccurrence of the negative percept and with sympathetic over arousal in cases in which interpersonal resolution and reassuring social support are lacking.

From a prevention perspective, uncertainty acceptance as an inevitable part of everyday life may increase resilience, support the availability of positive anticipations, and turn the anxiety disorder into the “confident brain.” This requires wider boundaries for creation of a negative perception and a higher threshold for negative expectation as suggested in the Drift Diffusion Model (114). This can allow the anxious individual to promote tolerance to uncertainty and reduce pathological motor activity resulting from threat perception of uncertainty. These wider boundaries (114) are associated with more confidence and less reward prediction error (115). We suggest that slowing down of automatic and habitual motor activity may support better excitation-inhibition balance within the junctions of sensorimotor circuits that recently have been shown to affect cortices and cognition as well as connectivity to the ANS.

By supporting anxious individuals during the COVID-19 situation to adopt wider boundaries for a decisive motor action as the first line of defense of treatment, those individuals may be able to perceive that the threat (infection and death of oneself and relatives) is not immediate and the reward (the pandemic will end as all previous pandemics did in history and the entire globe will get back to normal life) is out there in the future. These more adaptive perceptions may free the brain from pathological frequent motor reactions, which are part of the burden of anxiety disorders.

From a Control Theory view (84, 85), error signals emerge from the comparison of the actual value to the expected value and are organized as such. Faulty signals are disorganized, representing a faulty sensor of a controller, that is, neural noise and a pathological condition. In accordance with Powers (116) who suggested the term “reorganization” for describing the error correcting feedback loops process, we propose that an organized control system with systematic continuous error correction (healthy state) may turn into a disorganized control system which consists of sensors generating faulty signals (neural noise, pathological state) when faced with uncertain situations.

Only the comparison between faulty and reliable sensors could bring about an organized perception for a goal-oriented action to be taken. Setting a goal with the patient may work as an input to the cortex (109, 110), enabling cortical capacities for an overriding command and recruitment of sensorimotor regions. To support this view, it has been suggested in modeling the PCT, that higher regions act more rapidly than lower levels (109, 110). Thus, the cortex's ability includes the capacity for a prevailing command. Its representation is reflected in the transformed responses of the subcortical regions.

We note that assignment of goals, even in fantasy or imagination, is an input to the cortex and a goal is generated by the extent of the error signal (117). Lack of intention characterizes pathological conditions and a state of subcortical regions overtake resulting in incidental actions which are not clearly oriented. That is, selection and assignment of a goal and an intention in conditions of a disorganized system represents cortical transformation of the input-output balance to an organized state. We suggest that this way the cortex is able to activate a predominant descending command to reorganize the entire system including peripheral reactions as well as the upstream collection of ascending signals which were blocked in the disorganized state. According to Control Theory, the response signals from subcortical regions of the human control system to the cortical command represents the transformative dynamics of the command into appropriate\required signals of comparison. The subcortical regions are pre-programmed to respond to such a prevailing command. Pre-programing of subcortical regions develops early in fetal and infancy life through rostral to caudal and basal to alar trajectories while the developing control of the cortex over the brainstem is dependent on CST maturation (118, 119).

The faulty signals from a disorganized sensor distort external cues and overwhelm cortical input with internal disorganized signals thus preventing encoding and increasing subjective perception of uncertainty. In this sense revival of attachment links including past and present emotional securities may achieve the aim of computing a cortical command in conditions of disorganization.

The term “transforming command” in Control Theory represents in our view the human organism's capacity for fantasy and imagery. Accordingly, an assignment of a goal can be initiated in fantasy and imagery first. This is evident in the technique of guided imagery, in creative functions and in the therapeutic effects of attachment links' past intensities and sensations retrieval.

Beyond saving the CNS from faulty sensors, the human fantasy also encompasses the human capacity to go further and to transform the human control system into an accomplishing system rescuing the human organism from redundancy. Therefore, the preponderant cortical command is available to transform the human control system for negative and positive reasons, while the aligned actions to be taken, or results, in Powers's terms, are healthier when an error signal shows that the expected value is higher than the actual one. This condition may take the “confident brain” to further achievements while maintaining emotional survival in a stepwise multiple transformation of the human control system by a successive selection of goals. However, this positive and healthy process is also dependent on the error signal and internal reference standards for recruitment of cortical command and the dynamics of the subcortical transformed signal responses.

As Powers (89, 116) noted, error signals generated by higher regions comparing internal reference standards to the actual values of the perception allow the retrieval of past stored signals from lower regions. Thus, revival of attachment links may contribute to the restoration of organized error correcting and feedback top-down bottom-up signaling for a goal-directed action integrated by sensorimotor regions in a more organized manner, and may also turn pathology into a healthy process of growth further than the original aim of emotional survival in uncertain situations. This implies a shift in past personal programing, principles and systems, which organize the individual's principles, the highest modules in the PCT (94), and eliminates linear causality and forced negative predictions from any pathological condition. This proposition is in accordance with our view of the “anxiety spectrum,” a continuum ranging from “healthy anxiety” to pathology.

Providing increased security in close relationships during the COVID-19 situation may enhance excitation-inhibition balance within sensorimotor-driven cognitions of uncertainty. According to Bowlby (120) and Ainsworth (121), the sense of attachment security in close relationships (confidence that one can trust on relationship partners and that they will be available and supportive when needed) encourages relaxed exploration of new, unusual information and phenomena, and favors the formation of open and flexible cognitive structures. Being confident in their ability to deal with uncertainty, people who hold a sense of attachment security might be able to incorporate new information (sensory input) at the expense of temporary perplexity or confusion, reflecting “the confident brain.” Cognitive uncertainty might not generally threaten their sense of competence, lovability, and control. Rather, they might realize that perplexity, like other challenging experiences, is short-lived and can lead to greater mastery and broaden their sense of coherence and meaning (122) thus suggesting adaptive sensorimotor control.

In support of this view, there is extensive evidence showing that the sense of attachment security in close relations is closely related to heightened tolerance of uncertainty, lower levels of dogmatic thinking, and less rejection of information that challenges the validity of one's beliefs (123–125). Moreover, dozens of studies summarized by Mikulincer and Shaver (122) have consistently found that the sense of attachment security is associated with lower levels of anxiety and distress during and after exposure to stressors and less prevalence of anxiety disorders. Therefore, another line of defense to reduce anxiety in the COVID-19 situation might involve making contextually salient the close relations that a person feels securely attached to, or priming mental representations of attachment security in these relationships, using guided imagination exercises or structured autobiographical memories retrieval (126). This could potentially allow decisions on motor actions within more narrow boundaries and less prediction errors. The positive cognitions related to attachment security (“I'm worthy of love,” “Others are benevolent and trustworthy”) may afford decisive motor actions to protect the close others that provide emotional security (127, 128).

As a neurobiological background for the expected efficacy of these suggested treatments, which consider sensory input regarding the long-term sense of security to modulate habitual and automatic anxious reactions during times of change and uncertainty, we note that others found in fMRI sequential inference tasks multivariate patterns of activity representations that changed more rapidly during periods of uncertainty following a change in behavioral context. In motor cortex, this phenomenon was indicative of discontinuous change in behavioral outputs, whereas in visual regions, the same basic phenomenon was evoked by tracking of salient environmental changes. These results may provide a dynamic substrate for healthy learning that facilitates rapid disengagement from learned motor actions during periods of change (129). Furthermore, flexibility of boundaries in a context-specific manner, suggested here as a treatment technique, has been shown to buffer psychopathology during the COVID-19 situation (130). Taken together, retrieval of long-term solid sense of security and a build-up of boundaries' flexibility regarding a decision on an action to be taken, may increase the tolerance to uncertainty and provide a sense of certainty in controllable daily affairs with one's significant others, which in turn is thought to improve the status of individuals with anxiety disorders during the pandemic using the unique modulation of cognitions by sensorimotor regions.

## Conclusions

We suggest to extend the understanding of intolerance of uncertainty in anxiety-related disorders by the Sensorimotor-Cognitive-Integration-Circuit (SCIC). This assertion goes hand in hand with recent views suggesting that sensorimotor regions modulate cognitions. During the COVID-19 pandemic the issue of uncertainty gained much scientific attention. We add to this body of knowledge specific treatment suggestions based on Attachment Theory to shield the effects of the pandemic's uncertainties on emotional survival and on the tendency to collapse to anxious automatic and habitual motor reactions generated by negative cognitions due to lack of general control. According to our view, emotional security is generating emotional survival, a situation in which a human control system is organized. This in turn supports goal-oriented actions modulated via sensorimotor regions for the availability of inputs and outputs to the cortical perceptual functions and desired results of actions.

## Data Availability Statement

The original contributions generated for the study are included in the article/supplementary material, further inquiries can be directed to the corresponding author/s.

## Author Contributions

SGF wrote the initial draft of the paper and the revised versions. All authors contributed to editing various versions of the paper as it evolved and approved the final version.

## Conflict of Interest

The authors declare that the research was conducted in the absence of any commercial or financial relationships that could be construed as a potential conflict of interest.

## Publisher's Note

All claims expressed in this article are solely those of the authors and do not necessarily represent those of their affiliated organizations, or those of the publisher, the editors and the reviewers. Any product that may be evaluated in this article, or claim that may be made by its manufacturer, is not guaranteed or endorsed by the publisher.
